# Generation of twisted nanowires with achiral organic amphiphilic copper complexes†

**DOI:** 10.1039/c8ra09027k

**Published:** 2019-01-14

**Authors:** Carolin Isenberg, Eireen B. Käkel, Tobat P. I. Saragi, Peter Thoma, Birgit Weber, Alexander Lorenz

**Affiliations:** Macromolecular Chemistry and Molecular Materials (mmCmm), Department of Mathematics and Science, University of Kassel Heinrich-Plett-Straße 40 34132 Kassel Germany alexander.lorenz@uni-kassel.de; Department of Chemistry, Universität Bayreuth Universitätsstrasse 30 NW I 95440 Bayreuth Germany

## Abstract

Drying under solvent atmosphere (DUSA) was investigated as an experimental technique to generate self-assembled nanowires and needles from solutions of organic molecules under controlled conditions. Experimental observations of twisted nanowires are reported. These twisted nanowires were obtained by drying of solutions of achiral molecules under solvent controlled atmospheres: achiral, amphiphilic copper complexes were dissolved in an achiral solvent and these solutions were dried under controlled conditions. Two structurally related copper complexes were investigated. Microscopic investigations of the resulting nanowires revealed, and scanning electron microscopy confirmed: self-assembled twisted ribbons could be selectively obtained from one of these compounds. This behavior could be explained by comparing the ratio of the size of the head group and the overall length of the molecules. The occurrence of chiral filaments and chiral phases of nanosegregated filaments are rare in achiral compounds. The occurance of such twisted filaments is thought to be related to symmetry-breaking during a phase transition from liquid crystalline or lyotropic liquid crystalline phases to a nanosegregated phase. In the reported experiments, the concentration of a solution was gradually increased until crystallization occurred. The results clearly show how DUSA can be applied to investigate the capability of achiral substances to yield twisted filaments. Moreover, the investigated compounds had high-enough charge carrier mobilities, such that the twisted filaments obtained are candidates for self-assembled, intrinsically coiled nano-inductivities.

## Abstract 2

Drying under solvent atmosphere (DUSA) was investigated as an experimental technique to generate self-assembled nanowires and needles from solutions of organic molecules under controlled conditions.

## Graphical abstract

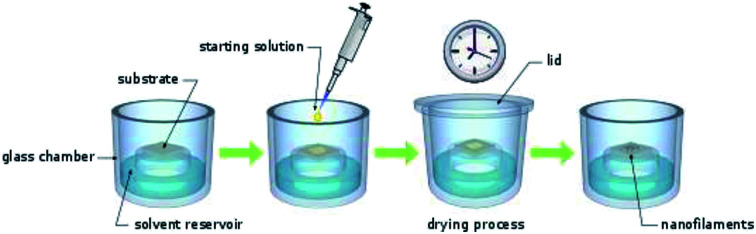

## Introduction

Chiral liquid crystals and solids of chiral compounds frequently show characteristic crystal structures with distinct point group symmetry and helical structures, such as in the chiral nematic phase and the helical molecular arrangement observed in chiral smectic C* liquid crystals.^1,2^ A crystal structure in solid or liquid crystalline phases consisting of chiral molecules shows a loss of local symmetry, which results in a different point group as the same crystal formed by achiral molecules (*e.g. D*_∞h_ in the achiral liquid crystalline nematic and smectic A phase changes to *D*_∞_ for the chiral nematic and chiral smectic-A phases, respectively).^1^ Rarely, chiral structures have been observed in achiral compounds: bent, amphiphilic molecules containing a diacetylenic moiety showed helical growth towards tubules^3–6^ and for bent-core or banana-like liquid crystals, the new phases B1–B7 (especially the helical nanofilament phase B4), could be identified.^7–9^ This unusual behavior was ascribed to a chiral symmetry-breaking mechanism during phase transitions.^9,10^ More examples where macroscopic chirality was detected in non-chiral compounds included a C3-symmetric benzene-1,3,5-tricarboxamide,^11^ which was substituted with ethyl cinnamate, spin casted dendritic zinc porphyrins,^12^ achiral bent core molecules,^13^ and Langmuir–Blodgett films of calcium arachidate.^14^ In lyotropic and thermotropic phases of amphiphiles, a reduction of water content or temperature could induce a phase transition from a fluent, liquid crystalline Lα phase to a flat gel Lβ phase, where the lipids were organized cooperatively.^15^ Here, the orientation of the hydrocarbon chains depended on the head group packing: while a small head group lead to parallel orientation of the chains with respect to the layer normal, a bulky head group resulted in a tilted arrangement (Lβ′). It has been assumed that this molecular tilt is the origin of the helical growth of the structure.^15,16^ The influence of the chain lengths on the crystal structure was determined for various iron complexes.^17^ In most cases, these complexes were arranged in a lipid layer-like molecular packing. The impact of the van-der-Waals (vdW) interactions (which lead to higher order in the lipid double layers) increased with increasing chain length of the hydrophobic tails.^17^ A lipid layer-like arrangement was also observed for one penta-coordinated iron(ii) complex with a short chain length of the ligand's hydrophobic tail (C8).^18^

**Scheme 1:**
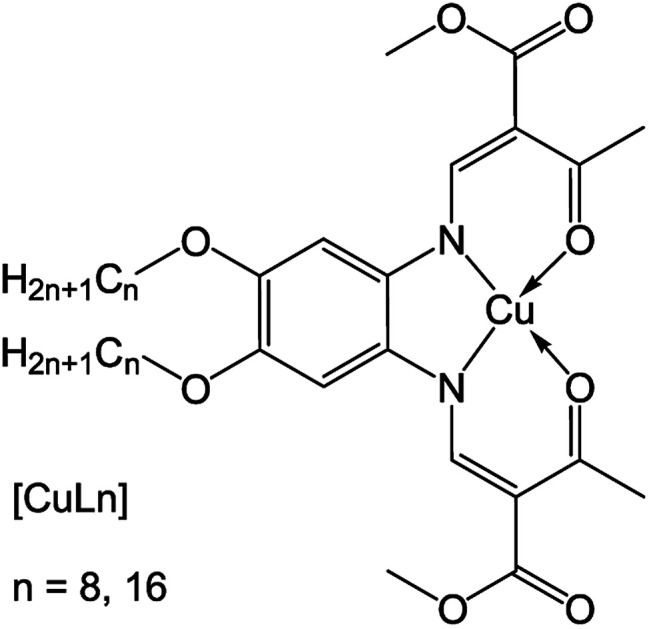
Structure of the amphiphilic copper complex with a Schiff base ligand (chain length of *n* = 8 and *n* = 16).

In this work, an alternative method is presented to obtain twisted nanowires from an achiral compound by a drying process controlled by solvent atmosphere. The aggregation properties of two copper(ii) complexes with amphiphilic Schiff base ligands were investigated. These species can be described as head-tail molecules with alkyl chains in the outer periphery of the Schiff-base ligand.^19,20^ Self-assembled filaments of two square planar copper(ii) complexes (Scheme 1) were successfully obtained by drying under solvent atmosphere (DUSA) – a useful, solution-based method to stimulate self-assembly of micro- and nanowires directly on a substrate surface, where no transfer process of the grown wires was needed.^21^ The two investigated complexes (CuLn) had the same core, but were synthesized either with a ligand that had an aliphatic tail with eight carbon atoms (CuL8), or sixteen carbon atoms (CuL16), respectively. Samples were investigated with scanning electron microcopy (SEM) and optical microscopy in combination with transmission electron microscopy (TEM). It was found that, depending on the experimental conditions, CuL16 self-assembled into well-separated, elongated nanowires (radius of 200 nm) with circular cross section and high aspect ratio. In contrast, the use of non-polar solvent atmospheres led to the appearance of twisted nanofilaments (flat nanoribbons) in CuL8, selectively. The charge carrier mobilities of nanowires and nanoribbons were investigated in several samples of both species. For these experiments, nanowires and nanofilaments were directly grown on field effect transistor (FET) substrates suitable for investigations with a semiconductor characterization system.

## Experimental

### Generation of nanowires

**Fig. 1:**
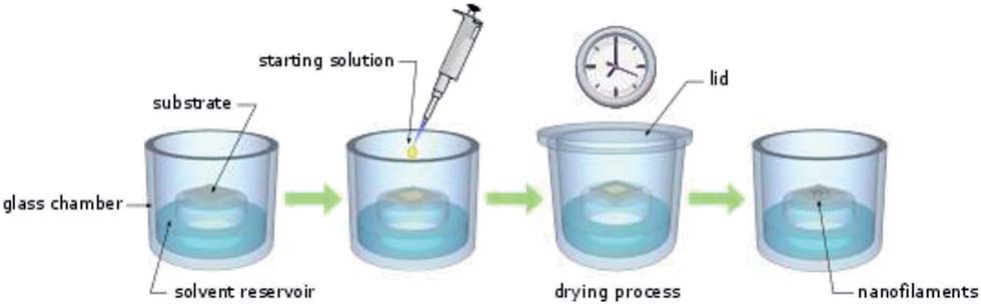
Schematic of the drying under solvent atmosphere (DUSA) technique. A glass chamber with reservoir and a central, protruded plateau was filled with solvent. A substrate was placed on top of the plateau. Once the atmosphere had saturated, the starting solution (0.01–1 μM in trichloromethane) was deposited onto the substrate. After waiting minutes to days, the droplet had dried and various self-assembled aggregation products (nanowires with different morphology) were found on the substrate surface.

CuLn micro- and nanostructures were obtained by applying the drying under solvent atmosphere (DUSA) technique.^21^ For this purpose, closeable, in-house assembled (glass blowing workshop) glass chambers were used. These glass chambers had a solvent reservoir and a protruded plateau with a diameter of 3 cm (Fig. 1). First, substrates (glass plates or silicon wafers) were placed on the plateau. Subsequently, the solvent reservoir was filled with a solvent, selectively, and the glass chambers were closed to obtain a saturated atmosphere. Various solvent atmospheres were investigated: hexane, cyclohexane (CH), methylcyclohexane (MCH), toluene, dichloromethane (DCM), ethanol, ethyl acetate, 1,2-dichlorobenzene (DCB), acetone, and *n*-methyl-2-pyrrolidone (NMP). Once the solvent atmosphere had saturated (after waiting one hour), a solution of the investigated species was deposited on the substrate. The species (1 mg) were first dissolved in trichloromethane (1 mL) and the solution obtained was subsequently diluted (starting-solution) to a concentration in the range of 500 μmol L^−1^ to 1 μmol L^−1^. For each experiment, various concentrations of the starting-solution were investigated. The lid of the glass containers was opened and a small (0.1–100 μL) portion of the starting-solution was deposited on top of the substrate with a micro pipet. Experience taught us, small sample volumes and deposition of a single droplet rather than a film can yield well-separated nano and micro wires during drying under solvent atmosphere rather than depositing films and higher sample volumes. After the lid was closed, the samples were investigated with the naked eye until trichloromethane had completely evaporated from the substrates. As expected, the time necessary to complete the drying process (drying time) depended on the droplet volume of the starting solution and the solvent atmosphere: approximately, one day in case of cyclohexanes and only several minutes in the case of DCM. Cyclohexane had a vapor pressure of 104 hPa (ref. 22) and DCM had a vapor pressure of 453 hPa.^22^ The drying time therefore apparently was not linearly depended to the partial pressure of the solvent atmosphere.

### Transistor preparation and characterization

Nanowires and nanofilaments were directly grown on the surfaces of FET substrates Fraunhofer IPMS (Dresden, Germany). A schematic of these substrates is shown in Fig. S1.† These substrates had a channel length (*L*) of 10 μm and a channel width (*W*) of 10 mm. The gate dielectric consisted of a SiO_2_ layer (230 ± 10 nm thickness). The source and drain electrodes consisted of an ITO layer (10 nm, adhesion layer) coated with an Au layer (30 nm). Prior to the deposition of the organic material, the FET substrates were cleaned with acetone and 2-propanol followed by oxygen-plasma treatment. Subsequently the cleaned substrates were exposed to hexamethyldisilazane (HMDS) to replace any surface hydroxyl-group by a nonpolar silanol group.

After the deposition (DUSA) of nanowires or nanofilaments, the FET samples obtained were transferred to a glove box (O_2_, H_2_O < 0.1 ppm) and placed in a home assembled sample holder and contacted with micromanipulators (Süss Micro Tec). Now, the samples could be analyzed with a semiconductor characterization system (Keithley 4200-SCS, equipped with preamplifiers for low-current measurements). Systematic current–voltage measurements were carried out at room temperature. Moreover, these experiments were conducted in a dark environment to avoid any unwanted exposure to light and thus measure the dark current, selectively. The data obtained was analyzed, for example the hole mobility was calculated in the saturation regime. More detailed information about the mobility determination in wire transistors is given in Fig. S2† and the corresponding caption.

The melting behavior of the two copper complexes was investigated with polarized optical microscopy. A small amount of each complexes in the solid phase (needles in both cases) was placed on a microscopy cover slip and heated in a Linkam hot stage (with silver sample holder). Both complexes showed a birefringent solid phase and were melted to form an isotropic liquid phase, respectively. Both compounds were heated and cooled repeatedly across the phase transition temperature of the crystalline phase to the isotropic liquid phase with slow heating- and cooling-rates of 1 °C per minute. A melting temperature of 180.5 °C was found in CuL8 and a melting temperature of 130 °C was found in CuL16.

In order to investigate the formation of lyotropic lamellar phases in dispersions of each copper complex in trichloromethane, both complexes were dispersed (three droplets of trichloromethane were added to 1 mg of the copper complex and the dispersions were sonicated) and these dispersions were studied with polarized optical microscopy: one droplet of the resulting isotropic, yellowish dispersions were placed on a microscopy slide and covered with a microscopy cover slip. Due to the presence of the cover slip, the evaporation process of trichloromethane was slowed down and phase transitions caused by locally varying concentrations of the copper complexes were easily observed. In such a sample, the concentration of the copper complex was locally varied due to the coffee stain effect (the concentration was high at the edges of the cover slip). At the initial concentration, both complexes formed isotropic, most likely micellar phases. Upon loss of dichloromethane, a phase transition (induced by loss of trichloromethane) to a lamellar phase and to the crystalline phase was clearly seen in CuL8 (Fig. S3†). The texture seen indicated a lyotropic Lα-phase. In contrast, no lamellar phases were seen in samples of CuL16.

### Synthesis of CuLn

All reagents were of reagent grade and used without further purification. All solvents were of analytical grade and used without further purification. Copper acetate monohydrate was used as received. Syntheses of the ligands H_2_L8 and H_2_L16 were performed analogously to procedures described previously.^17,20^

#### CuL8 (1)

Copper acetate monohydrate (72.4 mg, 0.36 mmol) and H_2_L8 (0.22 g, 0.36 mmol) were heated to reflux in ethanol (25 mL) for two hours. After cooling down to room temperature, the resulting brown colored precipitate was filtered off, washed twice with ethanol (15 mL) and dried. Yield 0.21 g (87%). Elemental analysis calcd. for C_34_H_50_CuN_2_O_8_ (678.33) C: 60.20H: 7.43 N: 4.13, found C: 60.26H: 7.67 N: 4.15; MS [ESI(+), 70 eV ]: *m*/*z* = 677 CuL8 100%, 452 [CuL8-C_16_H_34_] 65%; IR (ATR, *ν̃* cm^−1^): 2950, 2924, 2853 m (=CH, arom.), 1703 s (C=O, ester), 1603 s (C=O).

#### CuL16 (2)

Copper acetate monohydrate (0.25 g, 1.25 mmol) and H_2_L16 (1.05 g, 1.25 mmol) were heated to reflux in ethanol (80 mL) for four hours. After cooling to room temperature, the resulting brown colored precipitate was filtered off, washed twice with ethanol (15 mL) and dried. Yield 1.02 g (90%). Elemental analysis calcd. for C_50_H_82_CuN_2_O_8_ (902.76) C: 66.52H: 9.16 N: 3.10, found C: 66.51H: 9.52 N: 3.08; MS [DEI(+)]: *m*/*z* = 901 [CuL16-H] 100%, 452 [CuL16-C_32_H_66_] 39%; IR (ATR, *ν̃* cm^−1^): 2955, 2917, 2849 m (=CH, arom.), 1701 s (C=O, ester), 1604, 1583 s (C=O).

## Results and discussion

### Generation of nanowires

**Fig. 2:**
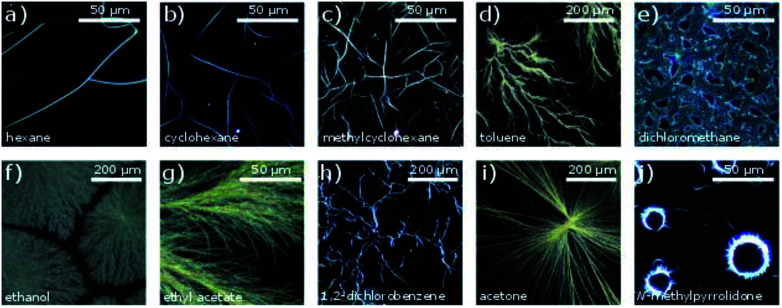
Optical micrographs of CuL8 aggregates fabricated from solution of the solvent trichloromethane (concentration 0.5 mM, volume 5 μL) in various solvent atmospheres (sorted in direction of increasing dipole moment) on glass substrates. Solvent atmospheres: (a) hexane, (b) cyclohexane, (c) methylcyclohexane, (d) toluene, (e) dichloromethane, (f) ethanol, (h) 1,2-dichlorobenzene, (i) acetone, (j) *N*-methylpyrrolidone.

**Fig. 3:**
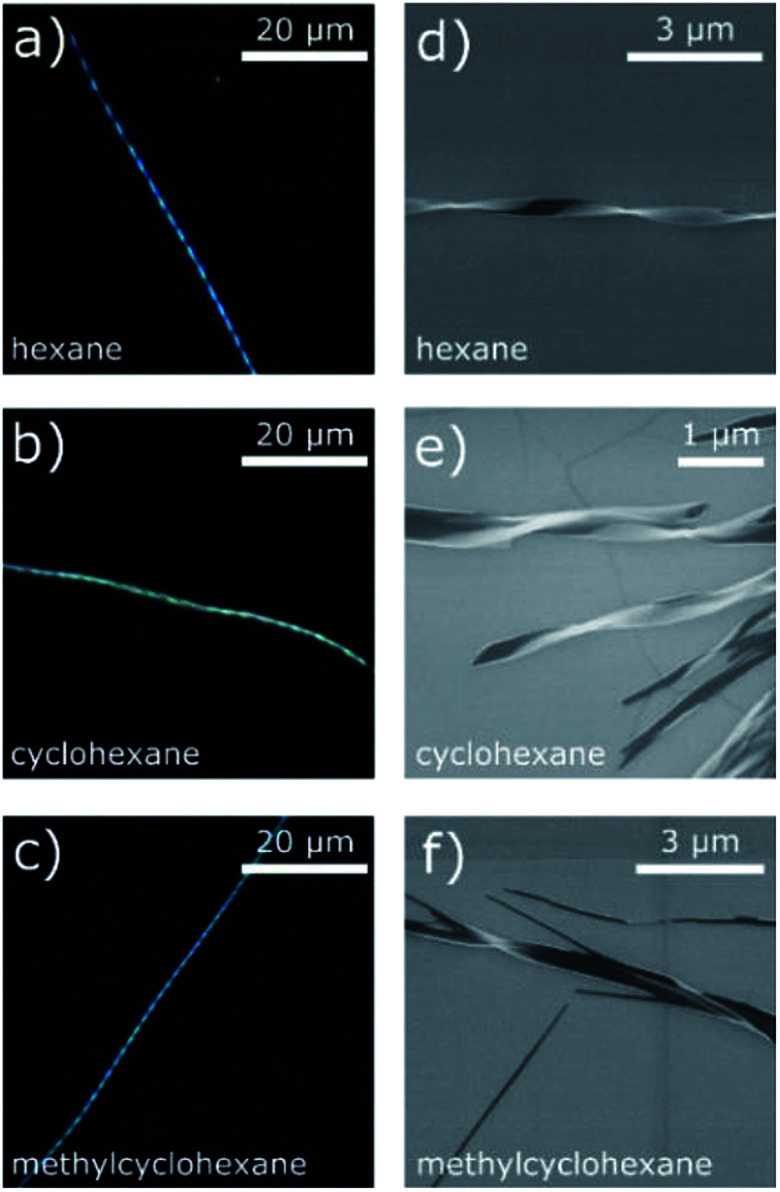
(a–c) Optical micrographs of twisted CuL8 nanowires obtained by DUSA: CuL8 0.5 mM in trichloromethane deposited on a glass substrate, evaporated in different solvent atmospheres on glass substrates. (d–f) Scanning electron micrographs of twisted CuL8 nanowires on silicon substrates.

The DUSA method was found to be a suitable approach to obtain micro- and nanowires from the studied compounds. The complex CuL8 was dissolved in trichloromethane. In order to achieve growth in a variety of self-assembled micro and nano morphologies, various solvent atmospheres were tested. Microscopic images of self-assembled dendrites and nanowires obtained are shown in Fig. 2. Twisted nanoribbons, obtained by carefully controlling the hydrophobicity of the solvents and concentrations used, were investigated with SEM (see ESI† for experimental details). The images obtained are shown in Fig. 3.

The observed morphologies were influenced by different factors: during the drying process, the molecules in the starting solution were interacting with one another, as well as with the substrate, the solvent and the atmosphere. Accordingly, the self-assembly was driven by intermolecular interactions such as π–π-interactions, dipole–dipole interactions and vdW interactions. In particular, π–π-interactions are known as promoter for one-dimensional growth.^23^ Indeed, single crystal X-ray structures of related copper(ii) complexes without alkyl chains show a high tendency of those complexes for π–π- or metal–π interactions that lead to stacks of parallel oriented molecules.^24^ These interactions can be influenced by the solvent and the solvent atmosphere. Results obtained in CuL8 by using various solvent atmospheres are shown in Fig. 2. The experiments revealed, both polarity and vapor pressure of solvent and solvent atmosphere are important for the self-assembly process. The DUSA-technique was used as a systematic approach to vary the growth conditions of self-assembly process systematically, which resulted distinct products for each investigated solvent atmosphere: one-dimensional structures (well defined, elongated, weakly crosslinked wires) were obtained for solvent atmospheres (hexane, CH and MCH) that possessed low dipole moments. For solvent atmospheres (toluene, DCM, ethanol, ethyl acetate) with medium polarity, two-dimensional structures like networks, dendrites and growth in domains were mainly observed. By use of solvent atmospheres (DCB and acetone) possessing higher dipole moments, the appearance of wires and needles increased again. For the highly polar NMP, needles in circular arrangements with a diameter of ∼30 μm were obtained. This specific behavior can be ascribed to a varied drying process: here (Fig. 2j), the solution formed small droplets on the substrate surface and due to the coffee stain effect, the material was mainly deposited at the edge of these droplets.^25^

Accordingly, solvent atmospheres with both high and low dipole moments led to the formation of elongated aggregates such as wires and needles, respectively. But the aggregates obtained were different: non-twisted filaments and needles were obtained with polar solvent atmospheres. In contrast, use of the solvent atmospheres hexane, CH and MCH (non-polar solvent atmospheres) led to the formation of twisted filaments, selectively. Such twisted filaments are seen in (Fig. 2a–c). Well-separated filaments were especially found after DUSA was completed in the locations near the edges of the deposited droplets, whereas at the droplet center, sometimes filament agglomerations were present. These fibers extended into branched wires and finally single wires were present in the peripheral region.

The twisted filaments obtained *via* DUSA with hexane, CH, and MCH solvent atmospheres were further investigated with SEM. The images obtained are shown in Fig. 3d–f. The SEM micrographs revealed a ribbon-like structure and Gaussian curvature. The Gaussian curvature was extracted from the SEM images, which revealed a mean width of 180 nm, a thickness of ≈50 nm and a mean pitch length of 1.8 μm (more information about the statistics of 25 randomly chosen wires is given in Fig. S4†). Both the left- and right-handed helical senses were observed. For hexane atmosphere (Fig. 3d), a larger number of isolated, single wires were seen than in the other atmospheres investigated. These twisted wires also possessed the shortest pitch lengths. The solvents CH and MCH (Fig. 3e and f) lead to aggregates and wire bundles. In such bundles, two or more wires could be twisted together (Fig. 3f). Additionally, the pitch length changed within one wire in both atmospheres.

**Fig. 4:**
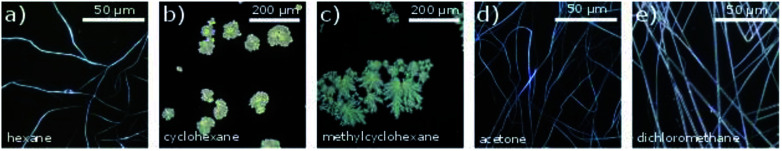
Optical micrographs of CuL16 aggregates grown by DUSA from samples solved in trichloromethane (concentration 0.5 mM) in various solvent atmospheres (sorted in direction of increasing dipole moment) on glass substrates.

By investigating copper complexes of the same ligand type with two different alkyl chain lengths, the twisting tendency of the nanoribbons could be understood. Structural investigations showed that the ratio of the size of the head group and the overall length of the molecule (which was determined by the length of the alkyl chains) significantly influences the self-assembly behavior. Longer alkyl chains can strengthen the vdW interactions and favor the formation of lipid layer-like arrangements.^15^ CuL16 showed no twisted wires in a variety of solvents atmospheres. The aggregation products for a selection of solvent atmospheres are displayed in Fig. 4. An overview of products obtained in all solvent atmospheres studied (sorted in direction of increasing dipole moment) is included in the ESI (Fig. S5†). Similar as in CuL8, one-dimensional structures were obtained in hexane atmosphere (Fig. 4a). In contrast, no helical filaments could be found. For CH and MCH, two-dimensional aggregates occurred (Fig. 4b and d), selectively. In exchange, long wires were obtained from acetone and DCM atmosphere (Fig. 4d and e). For DCM, a directed growth of the wires towards the center of the deposited droplet was seen, which was caused by contact line pinning and resulted in extended wires of several 100 μm length and diameters of 100 nm to 3 μm.

As mentioned in the introduction, lipid-layer arrangement twisted into chiral structures were observed.^11,12^ Both investigated amphiphilic complexes should also be able to form lipid-layer-like structures.^20^ Lipid layers with tilted molecular arrangement were discussed to play a crucial role in symmetry breaking towards assembly of chiral filaments.^15,16,26,27^ Therefore, the question arises why only CuL8 showed twisted ribbons, whereas from CuL16 no such structures could be obtained. The hydrophobicity of the longer alky chain might be one reason: In combination with stronger vdW interactions^19^ the aggregation towards two-dimensional growth could be enhanced in polar solvents. Nevertheless, CuL16 showed one-dimensional growth in acetone and DCM atmosphere, without the appearance of any twisted assemblies.

**Fig. 5:**
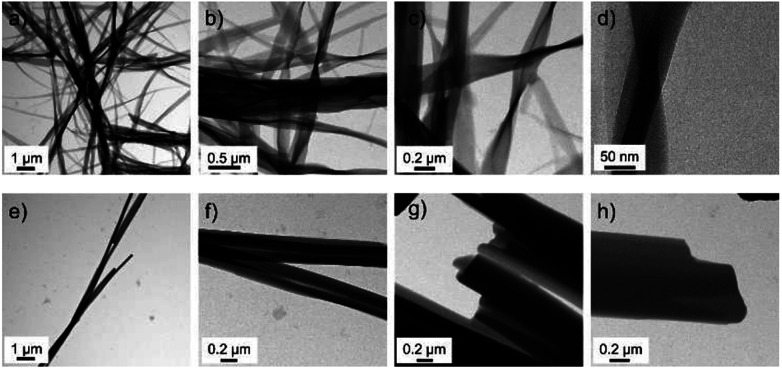
Transmission electron micrographs of nanowires obtained from dried isooctane solutions of (a–d) CuL8 and (e and f) CuL16.

In order to investigate the difference between the two complexes in more detail, samples were investigated with TEM (see ESI† for experimental details). TEM pictures of the two complexes obtained from dried isooctane solutions are compared in Fig. 5.

Again, twisted structures were observed for the complex CuL8 with the shorter alkyl chains while for the complex CuL16 linear rods were obtained. A closer inspection of those structures showed that in both cases, the structures were built from elongated, thin sheets. Apparently, for CuL16 (the longer alkyl chains and stronger vdW interactions) these sheets were thicker and more compact. Therefore, twisting was hindered. The origin of helical structures was assigned to a molecular tilt with respect to the bilayer normal,^15,16,26,27^ where the handedness was determined by the tilt direction of the molecules.^28^ Additionally, a tilt difference between both sides of the bilayer can further favor a saddle curvature.^12^ McIntosh showed that a bulky head group can cause molecular tilt, which leads to an increase of the vdW interactions between the hydrocarbon chains of amphiphiles.^29^ In CuL8 and CuL16 the head groups were identical, but for CuL8, the ratio of head to tail group was higher and the vdW interactions can therefore be considerably lower: CuL8 molecules have a more pronounced wedge or cone shape than CuL16 molecules. Therefore, a molecular tilt can occur,^26^ which leads to increased vdW interactions. The nonpolar solvent atmospheres hexane, CH and MCH (Fig. 2a–c and 3) were found to improve these hydrophobic interactions, selectively. The most likely reason was intercalation of these non-polar solvents between the alkyl chains.^15^ The investigated solvent atmospheres with higher dipole moments (Fig. 2d–j) led to non-twisted aggregates, here intercalation of the solvent between the alkyl tails of the amphiphiles during growth of the aggregates was less likely.

**Fig. 6:**
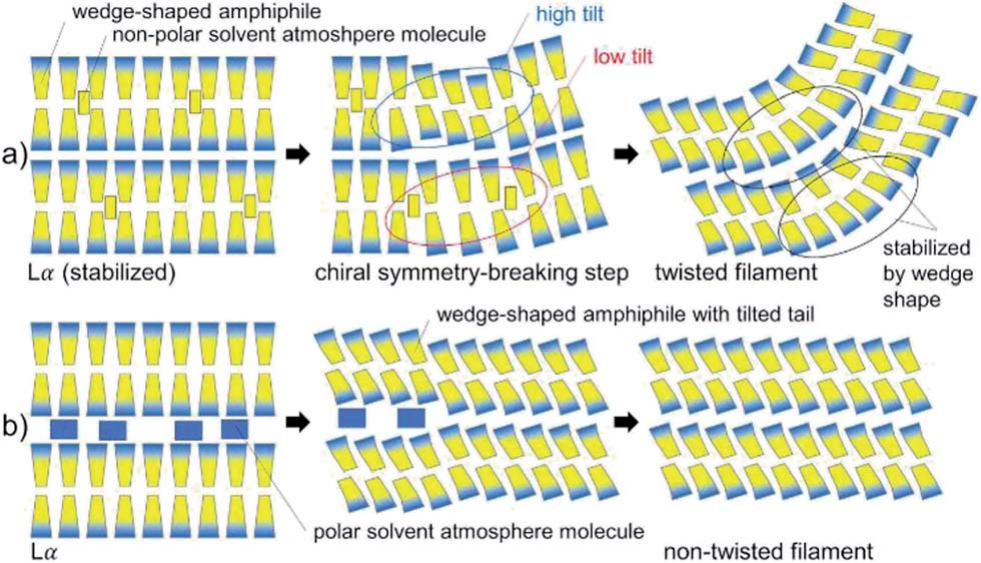
Schematic of the formation of twisted filaments in CuL8 upon loss of dichloromethane (drying). (a) Formation of a tilted filament by loss of non-polar solvent atmosphere molecules. (b) Formation of a non-twisted filament in a polar solvent atmosphere.

Both copper complexes formed needle- and filament-shaped crystalline powders. However, the single crystals are too small for single crystal X-ray structure analysis. Iron complexes with similar Schiff base ligands^30^ were seen to form crystals, where the molecules were arranged in bilayers with a molecular tilt. One can speculate about the chiral symmetry breaking mechanism during the formation of twisted structures in CuL8: the most important points given in the discussion of chiral symmetry breaking in the literature^15,16,26,27^ are presented in a simplified but straight forward schematic (Fig. 6) of the formation of a twisted structure in CuL8. In this model, it can help (but may not be ultimately necessary) to include the possibility of a tilt angle in CuL8 bilayers. It is well-known that the presence of tilt variations may induce chiral symmetry breaking. A lamellar Lα phase was seen in a CuL8 dispersion. This phase can be stabilized by a non-polar solvent atmosphere, where non-polar solvent atmosphere molecules are intercalated in the hydrophobic tails inside the bilayers (Fig. 6a). It has been discussed that wedge shaped amphiphiles have a higher tendency to form tilted phases than non-wedge shaped amphiphiles. Near the phase transition (induced by loss of dichloromethane) to the crystalline phase, loss of intercalated solvent atmosphere molecules may result in a locally tilted phase, with different average tilt angles as shown (Fig. 6a, step 2). This partially dried, titled filament will result in a tilted (and in two dimensions) twisted solid filament, once drying is completed. Here, the twist can be additionally stabilized by the wedge shape of the CuL8 molecules. In contrast to this, for polar solvent atmosphere molecules the loss of solvent molecules cannot induce tilt variances in the bilayers of amphiphiles, because polar molecules are intercalated between the polar head groups (Fig. 6b). The presence of a Lα phase and also the more pronounced wedge-shape in CuL8 as compared to CuL16 can thus explain why no twisted filaments were found in samples of CuL16. Anyway, it would be desirable to confirm the presented, deduced model with scattering methods in dispersions of both complexes and homologues.

In addition, different drying times of the droplets during the experiment can also have an influence on the resulting filament structures: in diacetylenic phospholipids,^4^ the symmetry breaking during multilamellar tubule formation was found to depend of the growth rate. Whereas the initial growth of the inner tubule produced left- and right-handed helical ribbons of roughly the same ratio, the following addition of outer layers showed uniform, right-handed sense. This observation was assigned to the growth rate: due to the slower growth process of the second layer, the tubule had more time to assemble in a thermodynamically ideal chiral structure. The chirality of the molecules was seen to influence the handedness of these chiral structures especially when the growth rates were slow.^4^ In analogy, the growth rate was also important for DUSA with the investigated copper complexes: both appearance and quality of single wires increased with increasing vapor pressure of the atmosphere: a high vapor pressure of the saturated solvent atmosphere resulted in slower drying-, and thus, slower growth rates.

### Transistor characterization

**Fig. 7:**
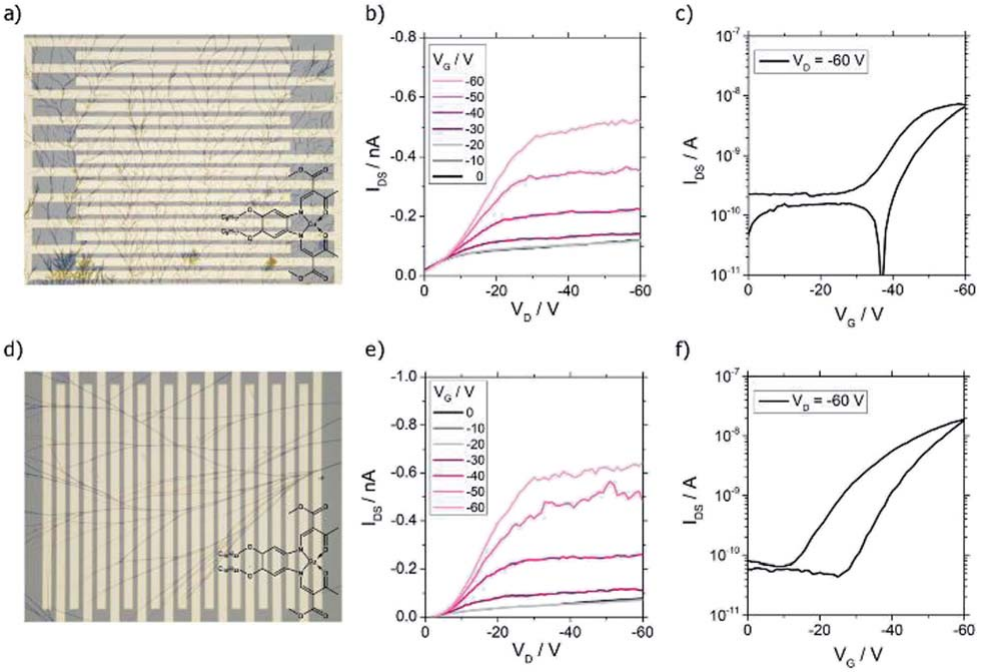
(a) Micrograph of a bottom contact field-effect transistor with CuLC8 wires fabricated by DUSA from the solvent trichloromethane (concentration 0.5 mM) in the solvent atmosphere acetone. The channel length (*L*) and a channel width (*W*) were 10 μm and 10 mm, respectively. (b) Output characteristic and (c) transfer characteristic of the CuL8 wire transistor in the hole transport regime. (d) Micrograph of a bottom contact field-effect transistor with CuL16 wires fabricated from solution of the solvent trichloromethane (concentration 2.8 μM) in solvent atmosphere dichloromethane. The channel length (*L*) and a channel width (*W*) were 10 μm and 10 mm, respectively. (e) Output characteristic and (f) transfer characteristic of the CuL16 wire transistor in the hole transport regime.

In order to investigate if samples with nanowires were candidates for nano- and microtructured transistors, the charge transport properties were investigated in bottom contact field-effect transistors. In this device geometry, it was feasible to deposit the one-dimensional structures in the last step of the transistor fabrication procedure avoiding damage of the tiny wires by another fabrication step. Due to different surface properties of the substrates, *e.g.* hydrophilicity and surface smoothness, the self-assembly products sometimes differed from the ones obtained on glass substrates. For CuL8, results for the solvent-atmosphere combination trichloromethane–acetone are shown (Fig. 7). To investigate the performance in dependence of the surface treatment of the transistor, substrates with and without HMDS were used. It was found that dendritic structures with different branch density were obtained, independent of the surface treatment (Fig. 7a). Anyway, devices without HMDS showed higher mobilities than devices with HMDS surface treatment (Fig. S6†). Probably, the higher performance seen in devices without HMDS surface treatment resulted from better contact of the conductive head group of the molecules with the polar gate surface.

The typical transistor characteristics are displayed in Fig. 7. Unipolar hole transport behavior was seen. The output shows a nonlinear relation at low drain voltage (*V*_D_) indicating a non-ohmic contact (Fig. 7b). This, and the high threshold voltage of ∼−40 V, were caused by an energy level mismatch of the gold electrode's work function and the unknown HOMO of CuL8 aggravated by a low contact of the wires with the gate. The transfer characteristic exhibited an on/off ratio of 10^2^ and small hysteresis. The hole mobility was calculated in the saturation regime according to eqn (S2.2) (see ESI).† For CuL8 in trichloromethane/acetone, the highest hole mobility measured was 7.1 × 10^−5^ cm^2^ V^−1^ s^−1^.

As shown in Fig. 6d, one-dimensional structures were obtained for CuL16 in trichloromethane/DCM. Use of transistor substrates with HMDS treatment lead to single wires, but only a few of the devices were covered by such one-dimensional structures, whereas most of the devices had void surfaces. Again, the non-polar surface was not beneficial for the self-assembly of the copper complexes. In contrast, without HMDS, branched wires were obtained in all samples (Fig. 7d). Also, the transistor performance was affected: without surface treatment, higher hole mobilities were reached (Fig. S6†). The output characteristics exhibited unipolar hole transport behavior with a small nonlinearity seen for small *V*_D_ (Fig. 7e). This was caused by an energy level mismatch of the Au electrode and the HOMO. This also led to high threshold voltages, which were slightly lower as compared to CuL8 transistors and were further decreased by HMDS surface treatment (Fig. S6†). The transfer characteristics show an on/off ratio of ≈5 × 10_2_ and a larger hysteresis (Fig. 7f). The highest hole mobility detected was 1.1 × 10^−4^ cm^2^ V^−1^ s^−1^ for CuL16 in trichloromethane/DCM.

## Conclusions

It was shown that the achiral copper complexes CuL8 and CuL16 self-assembled into one-dimensional wires and filaments, when applying the DUSA technique, where solvent atmospheres were systematically varied. The complex CuL8 (with a shorter alkyl chain) showed chiral aggregates such as twisted wires, ribbons, and filaments. In nonpolar atmospheres such as hexane, twisted ribbons were obtained (with both helical senses). These ribbons exhibited Gaussian curvature with a width of 180 nm, a thickness of ∼50 nm, and a pitch length of 1.8 μm. The origin of the twisted assembly was assigned to a higher head to tail ratio of this complex, as compared to CuL16. The wedge shape of the CuL8 molecules most likely resulted in a tilted bilayer arrangement during the growth *via* DUSA. Due to the wedge shape of the molecules, the vdW interactions were increased in this molecular orientation. Non-polar solvent atmospheres were found to further enhance these beneficial interactions.

Furthermore, we have applied the DUSA technique to obtain self-assembled nanowires on field-effect transistor test devices by using the two copper complexes investigated. The nanowire bottom contact devices showed unipolar hole transport properties with moderate mobilities of up to 7.1 × 10^−5^ cm^2^ V^−1^ s^−1^ and 1.1 × 10^−4^ cm^2^ V^−1^ s^−1^ for CuL8 and CuL16, respectively. DUSA is a promising technique to obtain interesting self-assembled products such as small wires and twisted filaments by varying solvent atmospheres.

## Conflicts of interest

There are no conflicts to declare.

## Supplementary Material

RA-009-C8RA09027K-s001
