# Supplementary material for: Generation of twisted nanowires with achiral organic amphiphilic copper complexes
Source: RSC Adv. 2019 Jan 14;9(4):1807–13. doi: 10.1039/c8ra09027k (PMC9059773; doi:10.1039/c8ra09027k)
Supplement: RA-009-C8RA09027K-s001 [file RA-009-C8RA09027K-s001.pdf]

Electronic Supplementary Material (ESI) for RSC Advances.
This journal is © The Royal Society of Chemistry 2019

Electronic Supplementary Information for

Generation of twisted nanowires with achiral organic amphiphilic copper complexes and their use in field-effect-transistors

by Carolin Isenberg,[a] Eireen B. Käkel, [a] Tobat P. I. Saragi,[a] Peter Thoma,[b] Birgit Weber[b] and Alexander Lorenz,[*a]

[a.] *Macromolecular Chemistry and Molecular Materials (mmCmm), Department of Mathematics and Science, University of Kassel, Heinrich-Plett-Straße 40, 34132 Kassel, Germany. E-mail: alexander.lorenz@uni-kassel.de*
[b.] *Department of Chemistry, Universität Bayreuth, Universitätsstrasse 30, NW I, 95440 Bayreuth, Germany.*

Characterization methods

Optical microscopy

Optical microscopy measurements were taken in transmitted light mode of the microscope from Leica DML.

Scanning electron microscopy

The scanning electron microscopy (SEM) measurement was performed using Hitachi S-4000 Scanning Electron Microscopy operating at an accelerating voltage of 10 kV. SEM samples were prepared on silicon wafers.

Transmission electron microscopy

Transmission electron microscopy (TEM) analyses were performed using a Zeiss LEO 922o (Carl Zeiss, 200kV). All samples were dissolved in isooctane (saturated solutions). A drop of the solution was placed on a copper grid with a lacey carbon film (LC200-Cu, Electron Microscopy Science) and allowed to dry for a few minutes.

Suppelemtary Figures

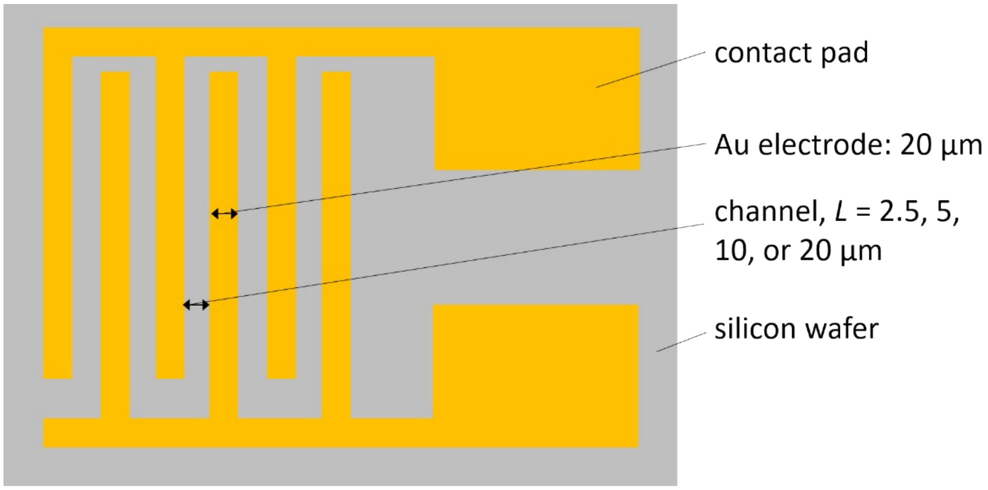

Fig. S1. Schematic of FET substrates purchased from Fraunhofer IPMS (Dresden, Germany). One Chip contains 16 separate transistor devices, for each channel length (20, 10, 5, 2.5 µm) four devices with a channel width of 10 mm. Onto the n-doped silicon wafer a 230 nm silicon dioxide layer was thermally oxidized. The contacts consist of a 30 nm gold layer with a 10 nm adhesion layer (ITO). The contact pads are 0.5 x 0.5 mm² wide.

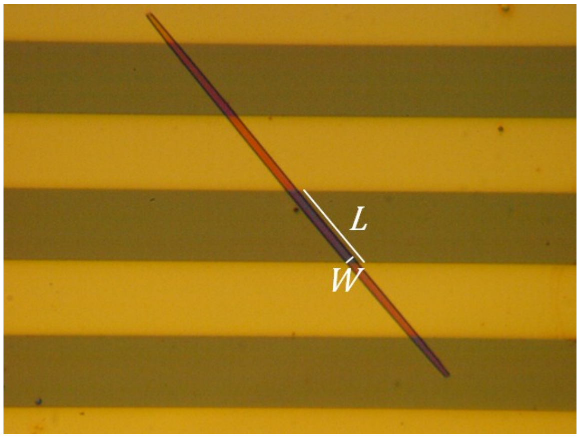

Figure S2. Optical determination of the channel length *L* and the channel width *W*.

The hole mobility μ was calculated in the saturation regime according to this equation:

$$I_D = \frac{W}{2L} C_i \mu (V_G - V_{th})^2 ,$$ Equation S2.1

With the drain current I_D, the channel length *L* and width *W*, the capacity C_i, the gate voltage V_G and the fitted threshold voltage V_{th}.

To calculate the mobility in a wire transistor, the channel width and length have to be determined for every wire crossing the conducting channel. The total current I_D arises from the current through every wire section I_j as in a parallel connection and is therefore represented by the sum of all partial currents $I_D = \sum_j I_j$ which results in equation S2:

$$I_D = \sum_j \left(\frac{W_j}{2L_j} \right) C_i \mu (V_G - V_{th})^2 .$$ Equation S2.2

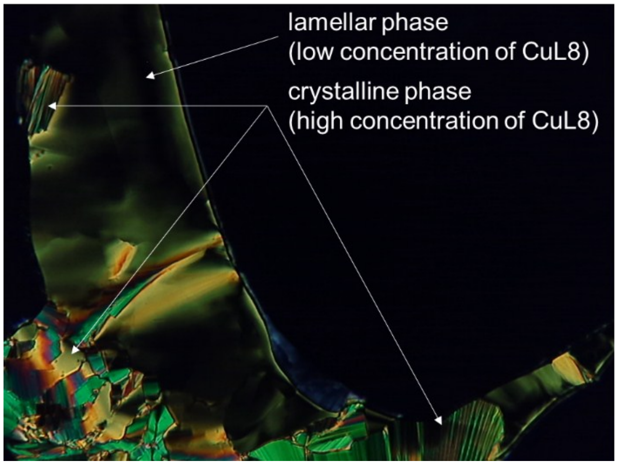

Fig. S3. Polarized optical micrograph (crossed polarizers, 20x objective lens) recorded in a partially dried dispersion of CuL8 dissolved in trichloromethane (three droplets of trichloromethane were added to 1 mg of CuL8 with a Pasteur pipette and the dispersion was sonicated). The yellowish dispersion was placed on a microscope slide and covered with a microscopy cover slip. The concentration of CuL8 was locally varied due to the coffee stain effect (the concentration was high at the edges of the cover slip). In a region near the edge of the cover slip, the phase transition of an isotropic dispersion (most likely a micellar dispersion of CuL8 in trichloromethane) to a lamellar phase and the growth of the crystalline regions was easily seen in the polarized optical microscope. Due to the presence of a cover slip, the evaporation was slowed down enough to observe these phase transitions. Crystalline regions and a region showing the texture of the lamellar Lα-phase are indicated.

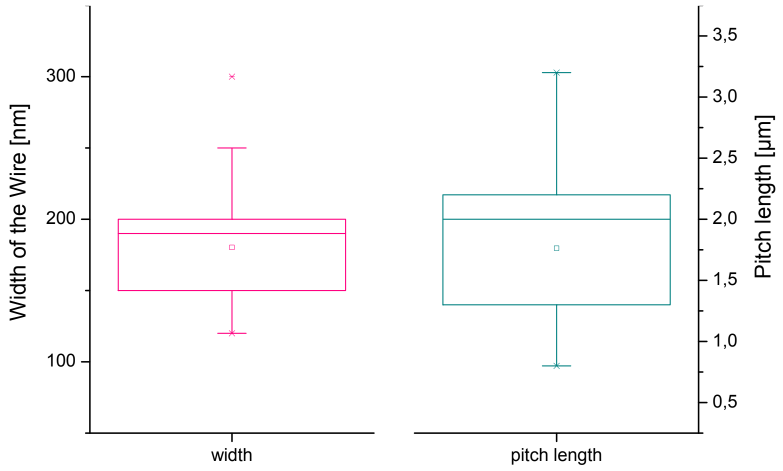

Fig. S4. Statistics of the wire width and pitch length measured by SEM pictures. Twenty-five twisted wires of CuL8 grown by DUSA from samples solved in trichloromethane (concentration 0.5 mM) in hexane atmosphere were chosen randomly. The mean width is 180 nm, while the values range from 120 to 300 nm. The width can vary by 30 nm within one wire. The pitch length ranges from 0.8 to 3.2 µm, with a mean value of 1.8 µm. Within one wire, a variation of 0.2 µm could be found.

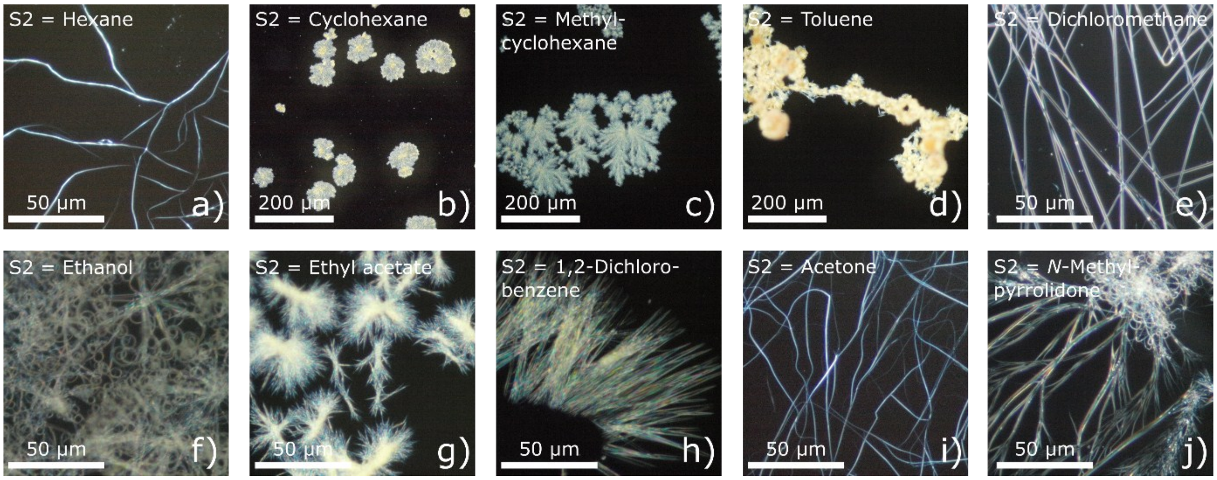

Fig. S5. Optical micrographs of CuL16 aggregates grown by DUSA from samples solved in trichloromethane (concentration 0.5 mM) in various solvent atmospheres (sorted in direction of increasing dipole moment) on glass substrates.

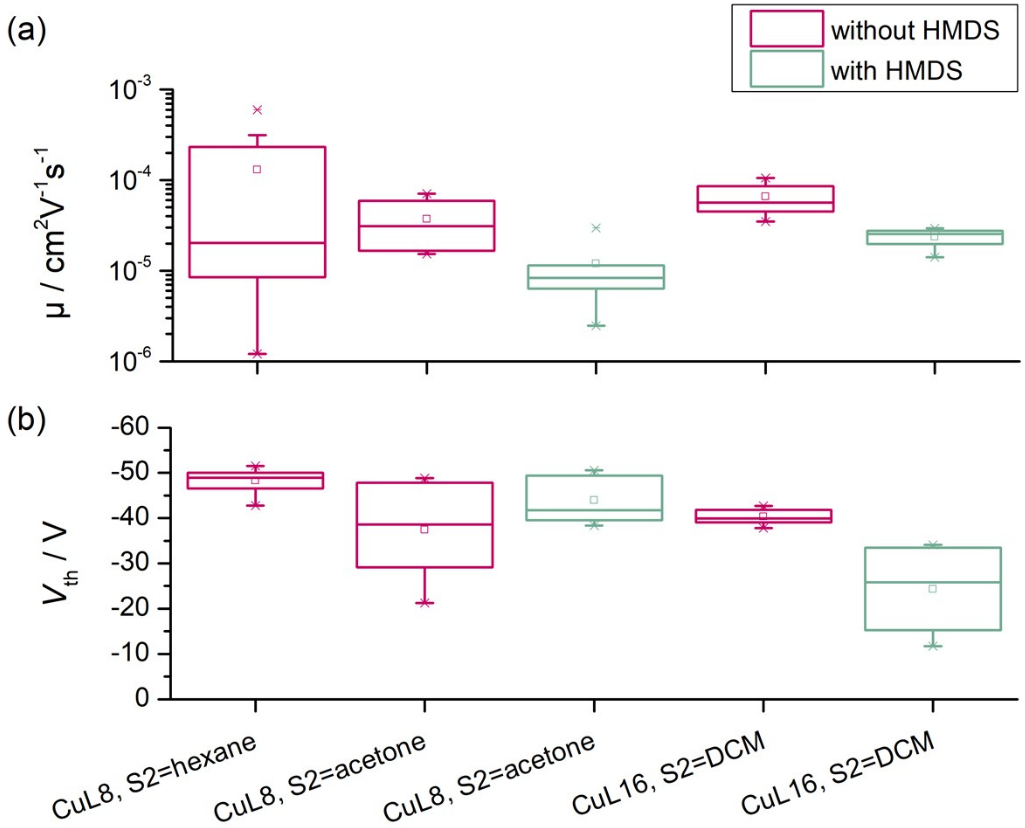

Figure S6. (a) Achieved hole mobilites μ for CuL8 and CuL16 wire transistors fabricated by DUSA from trichloromethane solution in different solvent atmospheres S2 on transistor templates from Fraunhofer IPMS with channel length (*L*) between 2.5 and 20 µm and channel width (*W*) 10 mm. To determine the influence of the adhesion promoter hexamthyldisilazane (HMDS) on the device performance two devices were prepared with and without HMDS under equal conditions. (b) Fitted threshold voltages V_{th}.
